# Priority setting in developing countries health care institutions: the case of a Ugandan hospital

**DOI:** 10.1186/1472-6963-6-127

**Published:** 2006-10-06

**Authors:** Lydia Kapiriri, Douglas K Martin

**Affiliations:** 1 Joint Centre for Bioethics, University of Toronto. 88 College Street, Toronto, Ontario, M5G 1L4, Canada; 2 Department of Health Policy, Management and Evaluation. University of Toronto Health Sciences Building, 155 College Street, Suite 425 Toronto, ONM5T 3M6, Canada

## Abstract

**Background:**

Because the demand for health services outstrips the available resources, priority setting is one of the most difficult issues faced by health policy makers, particularly those in developing countries. However, there is lack of literature that describes and evaluates priority setting in these contexts. The objective of this paper is to describe priority setting in a teaching hospital in Uganda and evaluate the description against an ethical framework for fair priority setting processes – Accountability for Reasonableness.

**Methods:**

A case study in a 1,500 bed national referral hospital receiving 1,320 out patients per day and an average budget of US$ 13.5 million per year. We reviewed documents and carried out 70 in-depth interviews (14 health planners, 40 doctors, and 16 nurses working at the hospital). Interviews were recorded and transcribed. Data analysis employed the modified thematic approach to describe priority setting, and the description was evaluated using the four conditions of Accountability for Reasonableness: *relevance, publicity, revisions and enforcement*.

**Results:**

Senior managers, guided by the hospital strategic plan make the hospital budget allocation decisions. Frontline practitioners expressed lack of knowledge of the process. *Relevance*: Priority is given according to a cluster of factors including need, emergencies and patient volume. However, surgical departments and departments whose leaders "make a lot of noise" are also prioritized. *Publicity*: Decisions, but not reasons, are publicized through general meetings and circulars, but this information does not always reach the frontline practitioners. Publicity to the general public was through ad hoc radio programs and to patients who directly ask. *Revisions*: There were no formal mechanisms for challenging the reasoning. *Enforcement*: There were no mechanisms to ensure adherence to the four conditions of a fair process.

**Conclusion:**

Priority setting decisions at this hospital do not satisfy the conditions of fairness. To improve, the hospital should: (*i*) engage frontline practitioners, (*ii*) publicize the reasons for decisions both within the hospital and to the general public, and (*iii*) develop formal mechanisms for challenging the reasoning. In addition, capacity strengthening is required for senior managers who must accept responsibility for ensuring that the above three conditions are met.

## Background

Because no health system, whether rich or poor, or privately or publicly funded, can afford to pay for every service it wishes to provide, *priority setting *is arguably today's most important health policy issue[1]. Much of the priority setting in a health system occurs at the so-called 'meso' level of policy making, which includes hospitals and health insurers. Yet, only a few studies have examined priority setting at this level, and these have focused on developed country institutions [2-5]. There is meager literature reporting actual priority setting in developing countries and it has focused on macro-level health reforms, health care financing or priority setting [6-10].

Developing country health systems can be strengthened by improving priority setting at the meso-level. This is because priority setting decisions contribute to the sustainability of strained pools of resources and have a direct impact on access to needed health services. Unfortunately, decision-makers in developing country healthcare institutions lack guidance with regards to priority setting [11]. As a result, priority setting in developing countries, such as Uganda, occurs by chance, not by choice [12].

Uganda spends 7.7% of its Gross Domestic Product of US$ 1,088 on health [13]. The country has a doctor to population ratio of 1: 25,000, a surgeon to population ratio of 1:30,000, hospital beds to population ratio of 0.9/1000, and an extremely high disease burden[14]. In attempt to meet the health needs and to maximize population health benefit, the Ugandan government has, (since 2000 AD) increased funding to primary care units relative to tertiary hospitals [15]. This has compounded the already difficult task of priority setting faced by decision-makers in tertiary hospitals. Hence, health managers in these institutions would benefit from having locally developed evidence-based strategies to guide their decision making.

This paper presents some of the findings from an endeavor to develop an evidence base to support decision makers in tertiary hospitals in developing countries. The approach used has been pioneered in developed country hospitals and employs the conceptual ethical framework of 'accountability for reasonableness' [16,17]. 'Accountability for reasonableness' is an explicit conceptual framework for legitimate and fair priority setting that has been used to evaluate and improve priority setting practices in health systems and health care institutions [18-22].

The purpose of this article is to describe priority setting in a Ugandan hospital and evaluate the description using a leading ethical framework, accountability for reasonableness, to identify good practices and opportunities for improvement.

## Methods

### Design

To describe priority setting in a hospital we used a qualitative case study. A case study is "an empirical inquiry that investigates a contemporary phenomenon within its real life context" [23]. The case study method is appropriate because priority setting in hospitals is complex, context-dependent and involves social processes. To evaluate the description, we used an explicit conceptual framework, 'accountability for reasonableness' (described below).

### Setting

**Figure 1:**
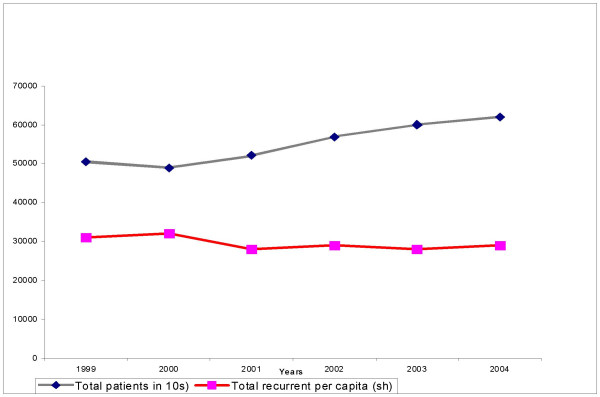
Total number of patients (10s) compared to releases on total reccurrent budget per capita.

The setting for this study was a 1,500 bed-publicly financed, tertiary teaching hospital in Uganda. Over the recent five years, the hospital has experienced a thirty percent increase in both inpatients and outpatients attendance (Figure 1). However, there has been a decline in funding to the hospital (in actual terms) e.g. from Ugandan Shillings 600 m in FY1999/2000 to Ugandan Shillings 500 m in 2000/2001, with serious implications on the availability of drugs and sundries [24].

### Sampling

We used a combination of theoretical and snowball sampling. The index respondent, the hospital deputy director, was identified by virtue of his involvement in priority setting. He identified subsequent respondents who were the leaders of the different clinical and support programs in the hospital. Those respondents identified subsequent respondents who they perceived to be key informants in relation to priority setting. Sampling continued until theoretical saturation was reached – that is, until subsequent interviews did not yield new data.

### Data collection

Data collection involved two data sources: i) in-depth one-on-one interviews with key informants, and ii) key documents.

We conducted 70 in-depth interviews with key informants involved in this case (14 health planners (including senior hospital managers, hospital accountants, chief pharmacist and the supplies officer), 40 doctors, and 16 nurses). These were identified using a combination of theoretical and snowball sampling [25]. Interviews were conducted using an interview guide having open-ended questions that were based on the conceptual framework described below (available upon request). However, the interviewer maintained an open stance and pursued emerging themes and sought clarifications as necessary. Respondents were asked to describe the priority setting process at the hospital management level, who was involved, what was considered, if decisions and rationales are publicized, if there opportunities for revision and mechanisms for enforcement. Interviews were audio recorded and transcribed.

The key documents reviewed included; minutes of the senior hospital management meetings, hospital budget estimates, and the Ministry of Health and hospital strategic plan.

### Data analysis

To *describe *priority setting, we used a modified thematic analysis: *First*, we read through whole interviews to identify general themes. *Second*, we identified the major concepts or ideas in specific chunks of sentences, and labeled them. An open and creative stance was sought throughout the process to facilitate identification of new ideas that related to different aspects of priority setting. *Third*, we grouped similar concepts together to form categories that were more precise, complete, and generalizable [25].

To *evaluate *priority setting, we compared the description against the four conditions of *Accountability for Reasonableness *to identify areas of correspondence, which were considered good practices, and gaps, which were considered opportunities for improvement.

We took three steps to ensure the validity of our findings. First, we interviewed respondents from different levels of the hospital management and professions. This maximized comprehensiveness and diversity. Second, we validated the interview data through the analysis of key documents. Third, the results were distributed to a number of respondents who confirmed the reasonableness of the findings (called a member check) [26].

### The conceptual framework

'Accountability for reasonableness' is a conceptual framework for legitimate and fair priority setting in healthcare institutions. It is theoretically grounded in justice theories emphasizing democratic deliberation [27,28], it was developed in the context of real-world priority setting processes and has emerged over the past five years as a leading framework for priority setting [16-22]. According to 'accountability for reasonableness', a legitimate and fair priority setting process meets four conditions: *relevance*, *publicity*, *appeals*, and *enforcement*-explained below.

*1. Relevance *condition: The rationales for priority setting decisions must rest on reasons (evidence and principles) that 'fair-minded' people can agree are relevant in the context. 'Fair-minded' people seek to cooperate according to terms they can justify to each other – this narrows, though does not eliminate, the scope of controversy, which is further narrowed by specifying that reasons must be relevant to the specific priority setting context.

*2. Publicity*: Priority setting decisions and their rationales must be publicly accessible – justice cannot abide secrets where people's well being is concerned.

*3. Revisions/Appeals*: There must be a mechanism for challenge, including the opportunity for revising decisions in light of considerations that stakeholders may raise.

*4. Enforcement*: There is either voluntary or public regulation of the process to ensure that the first three conditions are met.

'Accountability for reasonableness' helps to operationalize legitimate and fair priority setting in specific contexts, such as hospitals [1-4]. We used this framework to design our questionnaire and to analyze our data.

### Research ethics

This study was approved by the University of Toronto Office for Research Involving Human Subjects, the Uganda National Committee for Science and Technology and the hospital ethics committee. All participants provided consent for the interview. All data were kept confidential and anonymized.

## Results

The results section is organized in two subsections: *First *we describe priority setting according to the themes that emerged from our case study: *Second*, we evaluate the description using the accountability for reasonableness framework.

### 1. Description

#### The need for priority setting

Decisions makers in the study hospital encountered priority setting challenges everyday due to policy decisions at the national level which resulted in the hospital having a perpetual shortage of funds. For example, this financial year (2006/2007) the hospital submitted a budget estimate of 60 billion Shillings (US$ 32.4 Million), yet received Uganda shillings 25 billion (US$ 13.5 Million) which is approximately 30% of its budget estimates [29].

"... I think the problem is the shortage of funds from Finance...there is never enough money, so even though the directorate makes its budget, when the hospital gets it funds, there is always much less than what they require..."

According to our respondents, in previous years the hospital would spend beyond its budgetary limits and the Ministry of Finance would pay the deficits. However, in order to curb the national budget deficits, Ministry of Finance introduced budget ceilings beyond which the hospital cannot be funded; and line item financing as opposed to global funding which constrains the degree of flexibility in priority setting at the hospital management level.

#### Participants in priority setting

In the past, the hospital director and senior accountant submitted their budget directly to the Ministry of Finance. However, the introduction of Sector Wide Approaches (SWAP) – whereby donors support the health sector as opposed to vertical programs or institutions – has meant reduction in these direct negotiations. At the time of our study, all hospitals' budget negotiations occurred through the ministry of health.

Within the hospital, hospital managers have attempted to decentralized priority setting to directorates. However, due to various reasons (presented later in this paper) this has not been very successful and current priority setting still involves mainly the members of the senior management committee. The committee receives advice from the interim hospital board. They also receive input from the leaders of the directorates who should involve the frontline practitioners in identifying priorities within their departments. However the hospital managers felt that practitioners were reluctant to participate due to either time constraints, lack of interest, or power struggles.

"... But often you will find that it (involvement of frontline practitioners) doesn't happen like that, that's my disappointment as a manager. Because it involves letting go of power people don't want to let go, and actually even at the operational level also the head of the directorate doesn't want to let go. And at the departmental level, that head also doesn't want to let go to get his colleagues to bring their inputs."

This was corroborated by frontline practitioners who reported that they were not involved in the priority setting process. This lack of involvement contributed to their lack of knowledge of the priority setting process at the hospital management level. However, since they are daily confronted with patients and bear the direct consequences of the priority setting decisions, most of the frontline respondents thought they should be more involved in informing the hospital priority setting decisions.

Some of the departmental leaders that were involved in the process reported frustration since their concerns are often not addressed. For example, respondents from the department of pediatrics reported that they have repeatedly requested for cephalosporins – a broad-spectrum antibiotic, which is effective in treating most of the aggressive infections affecting their patients, but this has not been addressed due to lack of funds.

"...The problem is that sometimes you submit a proposal or a budget..., but not everything you have asked for are you able to get and sometimes what you have even put in the budget is not what is allocated to you. So, it can be frustrating..."

Participants reported that the public is involved through representation on the hospital board. One of the mandates of the board is to provide a link between the community and the hospital, however, since the board had not been officially instituted (at the time of the study), their effectiveness as representatives of the public could not be assessed.

#### What is considered?

Priority setting in the hospital occurs within the framework of the hospital strategic plan. *Formally*, there are pre-determined budget proportions whereby 50% of the budget is allocated to drugs, 30% to sundries, 10% to reagents and 10% to X-ray. These proportions are then further allocated according to a formula that is based on evidence and need (need was defined in terms of the number of beds per directorate, medical emergencies, and the patient load). The members of the senior hospital management team developed this formula, with input from the different departments.

However, respondents from the department of pediatrics and general medicine felt there was lack of adherence to this formula. They argued that according to the formula and the 'need' criterion, the department of pediatrics deserved to be prioritized since they receive almost 40% of the hospital emergencies. Since the department was not prioritized, these respondents thought that *informal *factors significantly influenced priority setting. They thought that departments whose leaders knew how to "lobby", "make noise", " quickly use up their resources", "make their case" are usually prioritized. As such, surgical departments seemed to receive disproportionately high priority.

"... You know resource allocation is political with a small " p". So sometimes you get departments or directorates which are either very vocal, and can argue their case very vehemently or very organized, in that once the money is available they know what exactly to do with it and they finish their part of the money and are ready to take the money from those who are not organized..."

"...*You see, as I told you that sometimes I may be getting things because I put a little bit of pressure on people, and I only leave when I have got what I want..."*

#### Communication of decisions

Various strategies are used to communicate priority setting decisions to staff members including meetings, circulars and an annual general meeting. The leaders of the various departments who are members of and should participate in the senior management meetings, are expected to communicate the decisions to the members of their departments. However, hospital managers doubted the effectiveness of this mode of communication, since many leaders fail to attend the meetings and those who attend did not communicate the decisions to their staff. In particular, departmental leaders with a dual role (of university professor and hospital manager) tended to value their roles and duties with the university more than their managerial roles at the hospital. This manifested as apathy in attending management meetings, with subsequent lack of understanding of the hospital planning management system, and lack of knowledge of the priority setting processes and decisions – which they should be communicating to their staff members.

The hospital management also tries to send circulars about key issues to all relevant departments. These are received and read by the frontline practitioners. However, several respondents expressed frustration since this form of communication is one way and provides no opportunity for feedback and dialogue.

The annual general meeting is convened for all the hospital staff. The hospital managers thought that this would provide an opportunity for staff and management to engage in direct dialogue over issues of interest. They, however, noted that attendance was still very disappointingly poor.

"...During the annual assembly information is given to the staff about how much money the hospital got, what the demands are, the priority areas of the hospital, this is to give them a general view. However the assemblies are very poorly attended by staff members. People don't seem to be interested..."

Mechanisms for communication of decisions and reasons to the public were less clear. The radio is occasionally used in response to crises, but it is not often used because of the costs involved. Respondents expressed mixed feelings about availing information about priority setting decisions to the public. Some respondents were weary of publicity and feared that the information, being too technical, would be misinterpreted by the public who may become more demanding. Others, however, felt that communication of decisions and reasons to the public-especially with regards to the resource constraints the hospital faces – would enable the public to have realistic expectations from the hospital and therefore deter the public from blaming the hospital management for the shortages of supplies within the hospital.

"...this information is not available to the general public. I must say that the public I think is fairly ignorant about the financial situation in the hospital. You know, there's a lot of blame placed squarely on foot of management for some of these things. But once somebody gets to what the facts on the ground are, people begin to change their perception about what they thought was a management fault..."

#### Dealing with disagreements

Frontline practitioners reported that they often disagreed with the priority setting decisions made at the hospital level, but were not aware of any formal mechanisms for challenging the decisions. In case of disagreements, practitioners usually write to, or verbally present their complaints to the senior management committee either directly or through the leaders of their departments. However, since they found that the management committee handled too many varying hospital related issues to address directorate specific complaints; practitioners often used the direct approach. They complained directly to the director of the hospital or his deputy who maintain an "open door" policy and could be accessed directly.

"... I actually often appeal through letters, directly to the Director, and you know, the Director then handles this on an individual basis, but I think it would be nice if there was a formal mechanism, or maybe if the formal mechanism exists, at least, for me to get to know it. I think it would improve also the running of the directorates if this actually happened on a regular basis, rather than when there was a crisis..."

Revisions of the priority setting decisions only occasionally occur, and are commonly in response to emergencies or crises. Usually this involves re-allocation of resources from one program to another, and is not popular. This lack of revision led people to question the usefulness of attending these meetings.

"...So there is that forum to which is the management committee and the all leaders of department are supposed to bring their comments.... In a way it could be like an appeal or a forum but you see what happens, if you come and complain, nothing is done, next month you come and complain... people lose morale they even cease to come. They just look at it as time wasting forum..."

### 2. Evaluation

#### Relevance

Resource allocation decisions were based on a complex cluster of both formal and informal factors. The formal factors identified in this study such as the strategic plan and the hospital's management formula, have been documented in other settings [3]. Informal factors, such as lobbying, exerting pressure on management, and reacting to crises, also played a role. Although respondents agreed on the relevance of the formal factors, there was lack of agreement about the relevance of the informal factors. Respondents who got what they wanted base on informal factors and mechanisms such as lobbying thought these should be considered relevant. This was because the director of the hospital, who makes the final priority setting decisions, maintained an open door policy, which meant that anyone who was dissatisfied with the priority setting decision had an equal opportunity to directly argue their case. However, since achieving the desired results depended on individual characteristics, such as one's ability to present a good case, those respondents who did not have the lobbying and advocacy skills felt that priority setting would be fairer if only the formal factors (and mechanisms) such as the strategic plan, evidence and need were the relevant reasons.

#### Publicity

There were attempts to communicate the decisions but not the rationales, to the hospital staff through meetings, and circulars, but these were not functioning well. In particular there was a breech in the flow of information from the management to the rest of the hospital staff.

The hospital lacked systematic mechanisms for publicizing priority setting decisions and the rationales to the general public. Publicity to the general public was through the radio and newspapers. However, because of the costs involved, this was ad hoc and often in response to crises. Some respondents thought it would benefit the hospital if the public had access to information about priority setting.

#### Revision/Appeals

There were no formal mechanisms for appealing the priority setting reasoning. The senior management meeting, which was thought to be the formal institution for appealing, was said to be less effective in revising the decisions once made. Some practitioners found that the informal mechanisms, such as complaining directly to the hospital director instead of going to the senior management committee, were more effective in getting them what they wanted. However, revisions to priority setting decisions was generally hampered by lack of resources and this failure to revise priority setting decisions by the management team was a source of frustration for front-line practitioners who often reacted by refusing to participate in the decision making processes. Respondents expressed the need for fair, clear, explicit, and more responsive mechanisms for appeals and revisions.

#### Enforcement

There was no mention by participants of any system to ensure that the above three conditions were satisfied. Mechanisms to ensure adherence to set criteria, follow up of the implementation of the decisions and evaluation of the impact of the decisions were also lacking.

## Discussion

To the best of our knowledge, this paper presents the first in-depth empirical description and normative evaluation of an actual priority setting process in a hospital in a low-income country.

Our study included the views of many stakeholders directly involved in decision making in this context. Absent from this group, however, were patients, families of patients, and members of the general public – who are also relevant stakeholders. Since the people who are involved in the decision making bring various considerations to the decision, the lack of identifiable stakeholders leads us to conclude that the full range of relevant considerations were not brought to bear in this case [30].

Priority setting decisions in this hospital were based on both formal and informal reasons. Most of the respondents considered the formal reasons such as those embodied with the strategic plan and the allocation formula to be relevant. These reasons coincided with those described in similar contexts in high-income countries [3]. The informal reasons, such as lobbying, have also been described in high income countries and were not universally accepted [31]. The lack of support for the informal reasons has also been documented at the macro-level in Uganda [32]. Therefore, the identified formal reasons, and already justified reasons such as the epidemiological data on disease prevalence and severity; costs, effectiveness of interventions, and equity [32,33]; should *first*, be evaluated for their ethical appropriateness, *then *debated by the full range of stakeholders to determine the most locally relevant reasons.

Decisions are available to the staff members of the hospital but not to the general public. According to some of our respondents, publicity to the general public would reduce misunderstanding, wrongful blame of the hospital management and increase public's confidence in the hospital. Publicity is also thought to improve priority setting by engaging all stakeholders in a kind of policy learning about appropriate limit setting decisions [22]. We found that there were efforts to publicize the decisions. However, the mechanisms employed were neither systematic nor effective. To improve publicity, the decisions AND reasons should be communicated at all management and departmental meetings, and publicized through a hospital newsletter or hospital webpage. Meetings should be participatory, and should involve: (i) Eliciting suggestions from participants when developing meeting agendas, (ii) Discussions and feedback. Since people are more motivated to participate if their recommendations are implementation [34], there should be clear action plans to follow up the implementation of the decisions made at these meetings.

Publicizing the decisions and the reasons to the general public maybe even more challenging given that most of the general public has low literacy and may require innovative approaches to communicating priority-setting decisions. Innovative, yet affordable approaches such as town hall meetings and print media should be explored. An annual general meeting involving the public would provide a platform for publicity. To ensure coherence, an acceptable level of detail and complexity should be determined through collaborations between management and public advisors and publicized in simple language with the use of illustrations. This information should be simplified for clarity, and presented in simple language with use of illustrations. Experiences from real life e.g. from New Zealand, and Tanzania [35,36], and from research settings in Uganda and Tanzania could be explored [37,38]. Although the radio would also be effective in publicizing this information, lack of resources may hinder its use. Should resources be available, optimal use of the radio would necessitate regular airing programs in the different dialects. The radio programs should be structured in such a way as to encourage public dialogue.

The concerns raised by some respondents that publicity may increase unrealistic public demands requires further investigation. However, research carried out in Uganda and Tanzania suggests that when people are provided with the necessary evidence, they are able to meaningfully engage in simulated limit setting decision-making [38,39]. These findings emphasize the need for systematic public education and provision of evidence on which decisions are based to the public.

With regards to the appeal/revisions condition, the hospital had ineffective formal and effective informal appeals mechanisms. Formal appeals mechanisms are deficient in many health care systems [17]. In which case, informal mechanisms such as lobbying, take precedence. Although they may be useful in getting a few "strong lobbyists" what they want, it is neither fair nor systematic and may be detrimental to the institution. The hospital should discourage informal mechanisms by refining the existing formal appeals mechanisms, making them explicit to the health practitioners and expanding the opportunity for appealing to other key stakeholders [5,19]. Information about these mechanisms should be publicized.

According to some of our respondents, direct lobbying of the hospital director, was thought to be an effective appeals mechanism because it gets people what they want. However, since some stakeholders may have privileged access to decision-makers, and some stakeholders may bring other 'back-door' techniques of persuasion to bear, this view does not align with a fair priority setting process [22]. In a fair process, an effective appeals mechanism features reason-based appeals by stakeholders and reason-based responses by decision makers. This give-and-take should be accessible to all stakeholders and perceived as consistent, transparent and open-minded, even in situations of disagreement about outcomes – i.e. when people do not get what they want. The managers of the hospital need to develop and publicize participatory guidelines for appealing and revisions, and communicate decisions and reasons in response to appeals.

There was lack of clear accountability mechanisms for decision making in the hospital. A similar finding was reported in a study of priority setting in a hospital drug formulary in Canada [4]. Clearly, the conditions of fairness cannot be met without deliberate direct action by hospital leaders [39]. Therefore, the hospital needs to explicitly determine who should be accountable for which aspect of priority setting. Furthermore, the leaders of the directorates should be held accountable for communicating to members of their departments through feedback mechanisms directly from the members of staff to management. Departmental meetings with a member from the senior management committee, (other than the departmental leader), in attendance, would facilitate this. The hospital management should ensure that either the head of department or the deputy is under the direct jurisdiction of the ministry of health. Then the hospital management would be certain of permanently having a representative who is directly accountable to them.

### Applicability

Accountability for reasonableness provides a framework for fair priority setting processes. Fulfilling the four conditions, especially where capacity and resources are constrained may be challenging, and may require making difficult trade offs, since a fair process as described in this paper may require resources which could be used elsewhere.

The authors recognize these constraints and recommend that when making these difficult decisions, in addition to considering the resources involved, decision makers, within their local contexts and realities, should also consider the justifications for implementing a fair process. *First*, acting fairly is the right thing to do. *Second*, it improves the legitimacy of the decisions. *Third*, some of the specific features of fairness, such as transparency and explicit reason-giving, may narrow the range of disagreement. *Fourth*, the fair process described here, which features stakeholder involvement, reason-giving, transparency, and responsiveness, helps to improve the quality of the decisions. An additional benefit of using an explicit framework, such as the one described here, is that it provides a common language for social policy learning that is accessible to all.

Should they choose to implement a fair process, decision makers need to consider what would be feasible considering their local realities. For some, this may mean starting off with implementing just one of the elements of a fair process, and adding the other elements as they progress; while others may develop innovative and less costly ways to implement all or some of the elements of a fair process.

### Limitations

The findings of this study may not be generalizable. However, generalizability was not our aim. This study provides an evidence base for improving priority setting in a hospital. These experiences may benefit other practitioners in similar contexts.

## Conclusion

We have provided a description of priority setting in a hospital in a low-income country and evaluated it against the leading framework, 'accountability for reasonableness'. The primary outcome is evidence – based recommendations to improve priority setting in this hospital and other similar contexts.

## Competing interests

The author(s) declare that they have no competing interests.

## Authors' contributions

LK and DK conceptualized the study. LK collected and analyzed the data. LK and DK conceptualized and wrote the paper. All authors read and approved the final manuscript.

## Pre-publication history

The pre-publication history for this paper can be accessed here:
